# Cementless Metaphyseal Sleeve Fixation in Revision Knee Arthroplasty: Our Experience with an Arabic Population at the Midterm

**DOI:** 10.1155/2020/5782853

**Published:** 2020-09-22

**Authors:** Abdulrahman D. Algarni

**Affiliations:** Department of Orthopedic Surgery, King Saud University, Riyadh, Saudi Arabia

## Abstract

**Objective:**

Metaphyseal sleeve (MS) fixation in revision knee arthroplasty (RKA) among Western populations has been reported with very encouraging outcomes. The aim of this study was to report our experience with the use of MS in RKA among an Arabic population. Clinical and radiographic outcomes and implant survivorship were reported at a minimum follow-up of 2 years and a mean follow-up of 4.1 years.

**Methods:**

A retrospective analysis was conducted on prospectively collected data of patients who underwent RKA with a MS in combination with a cementless stem (femoral or tibial). Range of motion (ROM) and Knee Society Score (KSS) were obtained pre- and postoperatively. Complications, occurrence of stem-tip pain, and implant survival were documented. Knee radiographs were obtained to evaluate the alignment and osseointegration or loosening of the MS.

**Results:**

A total of 52 sleeves (27 tibial and 25 femoral) implanted in 27 RKAs (27 patients) were included. The mean follow-up period was 4.1 ± 1.8 (2–7.5) years. Postoperatively, the ROM improved from 89.3 ± 9.2 to 106.3 ± 11.4 (*p* = 0.19) and the KSS also significantly improved, from 102.9 ± 35.6 to 130.2 ± 33.7 (*p* < 0.001). One patient (3.7%) developed heterotopic ossification, and another one (3.7 %) had a stem-tip pain on the tibial side; both were managed conservatively. One patient (3.7 %) sustained a fracture and required reoperation. None of the sleeves showed progressive radiolucent lines, and none required revision. The aseptic survivorship and overall survivorship at a mean of 4.1 years were 100% and 96.3%, respectively.

**Conclusion:**

MS provided successful midterm outcomes that were maintained in obese patients with different levels of constraint. Our series supports their use as a viable option in RKA.

## 1. Introduction

The extent of bone loss encountered in revision knee arthroplasty (RKA) is often underestimated on preoperative radiographs and may also increase considerably during the explantation of implants [1]. Bone loss is commonly classified according to the Anderson Orthopaedic Research Institute (AORI) system. AORI type 1 is an epiphyseal defect with an intact metaphysis, type 2 describes a damaged metaphysis involving 1 (2A) or both (2B) femoral condyles or tibial plateaus, and type 3 describes a deficient metaphysis that compromises the major segment of the condyles or plateaus [2].

Depending on the AORI type of bone loss, various methods have been proposed for reconstruction. These usually involve the use of stems, cementless or cemented, combined with several reconstructive options ranging from simple cancellous bone grafting and cement, metal augments and blocks, to total segment replacement of the distal femur and/or proximal tibia. The current literature does not support the superiority of either type of stem; both have obtained comparable outcomes in long-term follow-up studies [3]. While cemented stems offer immediate fixation, they are difficult to remove and have been associated with loosening and metaphyseal bone resorption due to stress shielding [4]. Likewise, cementless stems have an offset option but have been linked with diaphyseal cortical reaction and stem-tip pain [5]. Greene et al. have shown very promising results with the hybrid type of stem fixation, wherein the stem is press-fit in the diaphysis and cemented in the metaphysis [6].

The long-term survival rate in RKA is generally lower than that in primary total knee arthroplasty (TKA); hence, the need for improved fixation still exists [7]. Contemporary RKA systems, both cementless and cemented, increasingly focus on the metaphysis for defect augmentation and implant fixation [8]. Based on the successful experience of cementless metaphyseal fixation in total hip arthroplasty, cementless metaphyseal sleeves (MS) (DePuy Synthes, Warsaw, IN, USA) were introduced in the 1970s along with hinged RKA implants [9]. These are conical, stepped, and coated with titanium beads to encourage osseointegration.

Early to midterm studies of MS in RKA among Western populations have been reported with very encouraging outcomes [10–13]. We found one study among Chinese patients; the authors also reported excellent outcomes at the short-term follow-up [14]. To the best of our knowledge, similar outcomes among patients of Arabic ethnicity have not been reported. Therefore, the aim of this study was to report our experience with the use of MS in RKA among the Arabic population. Clinical and radiographic outcomes and implant survivorship were reported at a minimum follow-up of 2 years and a mean follow-up of 4.1 years.

## 2. Materials and Methods

Following the Institutional Review Board approval, we conducted a retrospective analysis of prospectively collected data of patients who underwent RKA with a MS (femoral or tibial) under the author's care between June 2012 and May 2018. The preoperative health status of the included patients was evaluated using the American Society of Anaesthesiologists (ASA) score. Details of patient demographics and clinical parameters are shown in Table 1. Patients were included until their latest follow-up date. Range of motion (ROM) and Knee Society Score (KSS) were obtained preoperatively and at the time of the postoperative follow-ups [15]. Complications (intra- or postoperative) and a survey of stem-tip pain were also documented. Implant survival, with failure defined as reoperation with the end point being aseptic loosening and reoperation for any reason, was recorded. Knee radiographs (anteroposterior (AP) in the weight-bearing position as well as lateral and skyline views) and hip-to-ankle (HTA) radiographs were obtained to evaluate the alignment and the presence of progressive radiolucent lines (RLs) at the bone-sleeve interface (level of osseointegration). Radiographic assessment was performed twice within an interval of 6 weeks by an independent observer. Loosening was defined as the presence of continuous two-millimetre RLs around the porous-coated part of the MS [16]. On the tibial side, the osseointegration was assessed in four metaphyseal zones (anterior, posterior, medial, and lateral). On the femoral side, the partially coated sleeve is coated only in the distal part, which is largely obscured in the AP view; therefore, osseointegration was only assessed in two zones (anterior and posterior) on the lateral view. RLs around the noncoated part of the MS or the stem were not considered signs of prosthesis loosening.

### 2.1. Surgical Technique

All patients received a revision prosthesis from DePuy Synthes. All RKAs included partially coated MS combined with the shortest cementless stem (length: 75 mm) on both the femoral and tibial sides. We aimed whenever possible not to exceed 14 mm stem-diameter on the tibial side to theoretically ease extraction of the stem and tibial tray through the sleeve if required later. In the case of a periprosthetic femoral fracture, a distal femoral replacement and a cemented stem, without femoral sleeve, were implanted. The DePuy Synthes RKA system offers different levels of constraint: the total condylar III-rotating platform (TC3-RP), which is a varus-valgus constrained implant, the Sivash-range of motion (S-ROM) Noiles, which is a rotating hinge, and the limb preservation system-distal femoral replacement (LPS-DFR), which is reserved for periprosthetic fractures. All the aforementioned femoral options with their respective rotating-platform inserts are compatible with the same mobile-bearing revision tibial tray and with the MS system. The choice of implant and level of constraint were determined by the amount of bone loss and ligamentous involvement. Details of the revision procedures and the components implanted are summarised in Table 2.

Adequate exposure was achieved through the medial parapatellar approach and release of the medial and lateral gutters. Quadriceps snip and/or tibial tubercle osteotomy were performed if further exposure was required. Following removal of the polyethylene (PE) insert, the components were explanted (femoral first) using a small oscillating saw and malleable osteotomes at the implant-cement interface to minimise additional bone loss. The cement was completely removed in all revisions, and thorough debridement with removal of the infected membrane was also performed in second-stage revisions. The AORI type of intraoperative bone loss is summarised in Table 3. The tibial and femoral medullary canals were then reamed until cortical diaphyseal contact was achieved. The tibial metaphysis was then prepared using a broach in consecutive sizes to accept the sleeve, using the trial stem to ensure that the broach was centred within the canal. We sometimes undersized the diameter of the trial stem by one to ease the broaching. The sleeve was correctly sized when it had a tight fit with rotational stability. On the femoral side, we aimed to ream and broach the canal as posterior as possible to build up the posterior femoral condylar offset (PFCO) and address the large flexion gap. The rotationally stable femoral sleeve needed to restore the joint line was selected. Femoral condylar augments (distal and posterior) were used as appropriate to address epiphyseal bone loss. Soft tissue release was performed as appropriate to adequately balance the knee, and a trial reduction was carried out. The definitive prosthesis was then assembled on the back table, taking care to match the same rotational orientation of the trial component. Cement was applied to the undersurface of the tibial tray and the femoral component (FC), taking care to avoid the porous-coated part of the MS.

### 2.2. Statistical Analysis

Data were analysed using SPSS statistical software version 22 (IBM Corp., Armonk, NY, USA). Pre- and postoperative ROM and KSS score values were compared using the dependent-sample Student's *t*-test. The intraobserver reliability (IOR) of radiographic evaluations was assessed using Cohen's kappa analysis. The correlation between the AORI type and the outcome variables was assessed with the chi-square analysis or Fisher's exact test, as appropriate. Implant survival was assessed using the Kaplan–Meier analysis. A *p* value of <0.05 was considered statistically significant.

## 3. Results

We identified a total of 52 sleeves (27 tibial and 25 femoral) in combination with 52 cementless stems implanted in 27 RKAs (27 patients). The mean follow-up period was 4.1 ± 1.8 (2–7.5) years. One patient died (of an unrelated cause), and another was lost to follow-up at 5 and 3 years after the index revision, respectively. Both these patients were included until their latest follow-up. The index revision was first time revision in 25 (92.6%) knees and second time revision in 2 (7.4%) knees.

**Figure 1:**
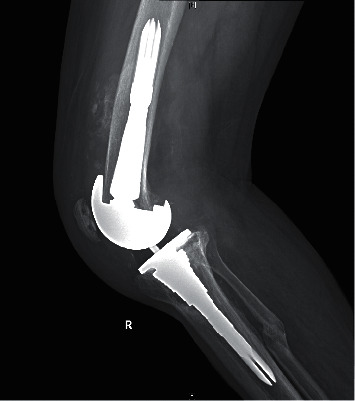
Lateral radiograph of the right knee showing the heterotopic ossification.

At the most recent follow-up, the preoperative ROM improved from 89.3 ± 9.2 to 106.3 ± 11.4 (*p* = 0.19). The KSS also significantly improved, from 102.9 ± 35.6 to 130.2 ± 33.7 (*p* < 0.001). There was no significant correlation between the AORI type and the functional outcome, the level of osseointegration, or implant survival (*p* = 0.51, *p* = 0.23, and *p* = 0.3, respectively). There were no intraoperative complications. One patient (3.7 %) (man; 70 years; body mass index (BMI), 31.3 kg/m^2^, ASA 3) postoperatively developed heterotopic ossification (HO) at the anterior surface of the distal femur (Figure 1). At his latest 3-year follow-up, he maintained reasonable ROM (5–90), and the HO did not interfere with his function; thus, excision was not required. One patient (3.7 %) (woman; 72 years; BMI, 42.4 kg/m^2^, ASA 3) had a stem-tip pain on the tibial side for 5 months postoperatively; however, it completely resolved spontaneously thereafter.

The IOR of radiographic assessment showed substantial agreement (0.79). Postoperative HTA radiographs revealed optimal (±3 of neutral) mechanical alignment (−1.7 ± 2.9) compared to preoperative values (−8.9 ± 4.1) as measured using the hip-knee-ankle angle. One (3.7%) tibial sleeve had RLs <1 mm in 2 zones but did not progress. None of the sleeves showed progressive RLs in the porous-coated part, and none required revision.

**Figure 2:**
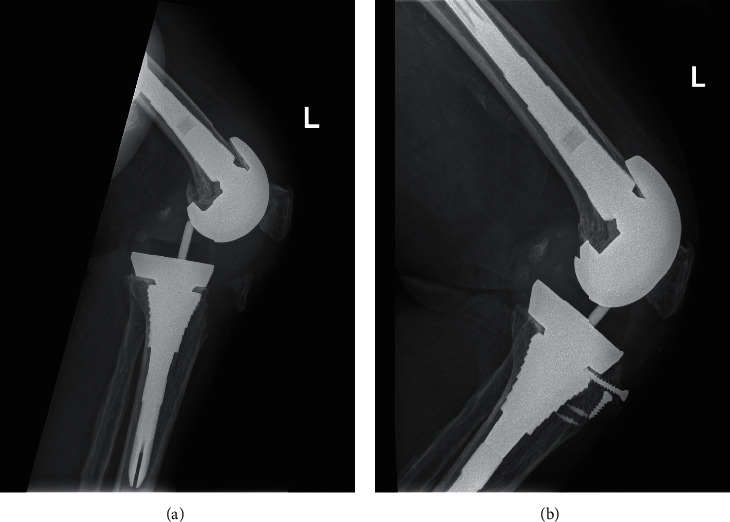
(a) Lateral radiograph of the left knee showing the posttraumatic tibial tuberosity avulsion fracture and the subluxated prosthesis. (b) Lateral radiograph of the same knee at the most recent 2-year follow-up showing a healed fracture.

One patient (3.7%) (woman; 76 years; BMI, 38.9 kg/m^2^, ASA 2) underwent reoperation 2 years after her index revision. She fell and sustained an avulsion fracture of the tibial tuberosity with a subluxated prosthesis in association with an ankle fracture of the contralateral side. She was successfully treated; the tuberosity fracture was reduced and fixed with screws and suture anchors, the prosthetic knee was reduced, and the liner was exchanged. Radiographs after the fall and at the most recent 2-year follow-up are shown in Figure 2. Implant survivorship was therefore 96.3% at 4.1 years, with the end point being reoperation for any reason, and 100% survival at 4.1 years, with the end point being reoperation for aseptic loosening of the MS.

## 4. Discussion

The main findings of the present study were the excellent rate of functional outcomes, osseointegration, and survival rate. The aseptic survivorship and overall survivorship at a mean of 4.1 years were 100% and 96.3%, respectively.

Bone loss management and secured implant fixation are the two main challenges encountered in RKA [17]. The concept of zonal fixation has been recently proposed to assist in securing the fixation of revision implants [18]. It defined three potential zones for fixation: zone 1 (epiphysis), zone 2 (metaphysis), and zone 3 (diaphysis), and recommended implant fixation in at least 2 of these 3 relevant zones. The MS combined with the stem has enabled implant fixation in two zones (2 and 3), even if the epiphysis is severely compromised. MS enabled direct fixation of the implant and bone loss management in a single step. The osseointegration potential of the sleeve is enhanced by metaphyseal bone loading, which reduces stress shielding, and by the mobile-bearing interface, which reduces the shear forces through the sleeve [19,20]. Porous-metal cones, such as the trabecular metal system, tantalum (Zimmer Biomet, Warsaw, IN, USA), can also address metaphyseal bone loss [21]. As opposed to sleeves, the cones will also need diaphyseal fixation, usually with a cemented stem that also allows fixation of the stem to the cone until the cone is osseointegrated. Therefore, an additional interface exists between the cone and prosthesis. It remains to be seen if this could have a significant impact on long-term fixation. Cones have the advantage of being a more flexible tool to address bone loss, independent of the type of the revision system implanted in contrast to the MS, which are implant-specific, as they are a fixed part of the prosthesis [22]. Midterm studies regarding the use of cones in RKA have shown very encouraging results in terms of functional outcomes, osseointegration, and the aseptic loosening rate that were comparable to those achieved with MS [23,24]. Further long-term studies are needed to determine the superiority of either method.

There is a paucity of studies comparing the MS with a standard diaphyseal stem-based RKA prosthesis. In a recent study by Lai et al., the authors reported comparable clinical and radiographic outcomes of the MS and the constrained condylar knee (CCK) prosthesis (Zimmer Biomet, Warsaw, IN, USA) at a mean of 2-year follow-up [25]. Studies have shown successful outcomes of MS in RKA with significant improvement in functional scores and excellent rate of osseointegration [26–28]. Implant survival rates of 94% to 100% have been reported at the midterm follow-up, with the endpoint being aseptic loosening [29,30]. Bloch et al. recently reported a large series of 227 patients (319 RKA) using the MS (319 tibial and 146 femoral) with a mean follow-up of 7.6 years [30]. The study demonstrated excellent implant survivorships of 99.1% at 3 years, 98.7% at 5 years, and 97.8% at 10 years. None of the MS had progressive RLs, and none were revised for aseptic loosening in this study. Similarly, another recent study by Kim et al. which included 92 patients (93 RKA) with a mean follow-up of 6.3 years also demonstrated satisfactory results. The authors reported average improvements of the Western Ontario and McMaster Universities Osteoarthritis Index (WOMAC) from 55 preoperatively to 9 postoperatively and the Subjective Satisfaction Score (SSS) from 2 preoperatively to 8 postoperatively (both *p* < 0.05) [29]. All patients in their series demonstrated optimal osseointegration at the final follow-up. Our study also demonstrated similar improvement in KSS, osseointegration, and implant survival at a mean follow-up of 4.1 years. In the present study, the cohort consisted predominantly of female patients (81.5%) who were obese (BMI = 37.68.1 kg/m^2^; Grade 2: moderate obesity) with the ASA 2-3 score. It seems that obesity did not negatively impact our outcomes; we had successful outcomes among our patients with no case of aseptic loosening at the midterm similar to that published among Caucasians. Comparing our outcomes to those in other patients of Arabic origin was limited by the lack of studies in this context. To the best of our knowledge, ours is the first report on the use of MS in RKA of Arabic patients. The female predominance in our cohort has been observed in other series and was attributed to postmenopausal osteoporosis [31,32]. We believe that this is a reflection of the higher percentage of primary TKA in our female patients as compared to their male counterparts.

Another advantage of the MS we experienced was the ease of intraoperative reconstruction of the PFCO, although postoperative measurement of the PFCO was not analysed in this study. The most frequent FC used in our series was size 2, followed by size 1.5 (both are the smallest sizes) because of the documented smaller bone geometry in the knees of people of Arabic ethnicity in contrast to that in Caucasians [33]. Keeping the femoral canal reaming and broaching as posterior as possible and using the largest stable femoral sleeve to restore the distal joint line combined to the shortest stem to stay distal to the femoral bowing allowed us to address a large flexion gap without reverting to larger FC, which is not always possible in patients with small bone geometry, as in our study. It may also cause component overhang with the risk of soft tissue irritation. Wirries et al. reported that they could restore the distal joint line at a mean of 0.36 mm lower to the anatomic level using the MS [34]. We are not aware of any study comparing the amount of PFCO obtained using the MS in contrast to an offset stem. However, it should be remembered that the offset stem sometimes will have only press-fit more proximally, leading to anterior shift of the FC, resulting in a larger flexion gap. Consequently, a higher PFCO is required in contrast to the MS that fits in the metaphysis and distal diaphysis. Using this technique together with the thick tibial tray options (15 and 25 mm) provided by this system and meticulous soft tissue balancing obviates the need for thick PE inserts in most patients in our series (median PE insert was 12.5 mm).

Although MS has advantages, there are some concerns that need to be addressed. As the removal of the osseointegrated sleeve is challenging, a fully porous-coated sleeve should be reserved for selected cases. In addition, using a tibial stem not more than 14 mm in diameter is recommended to theoretically allow explantation of the stem through the osseointegrated MS in case of further revision. In our series, we exclusively used partially coated MS and avoided tibial stems greater than 14 mm when possible. Fortunately, we were not required to remove any osseointegrated MS to date. Intraoperative fracture during metaphyseal broaching is also a potential complication [8]. This complication was not observed in our study. Another concern is stem-tip pain. The rate of stem-tip pain in the current study (3.7%) was comparable to the findings of Graichen et al. (3.2%) and Martin-Hernandez et al. (2.2%) [35,36]. Interestingly, the stem-tip pain in our patient spontaneously resolved within six months after the procedure. It is unknown whether MS osseointegration contributes to pain subsidence. Although it may be possible to use the stemless sleeve construct, it has remained our practice to add the shortest stem to the MS in all cases to achieve 2 zones fixation. The initial stability offered by the stem until the MS is fully osseointegrated and the benign course of the stem-tip pain in our series are also in support of the stem use. Therefore, we recommend the use of the shortest (length: 75 mm) cementless stem with any implanted MS. Nadorf et al. advocated the use of a short tibial stem to reduce micromotors of the tray and sleeve and to reduce proximal stress shielding, which occurs with no stem or long stem use, respectively [19]. Although Thorsell et al. described good midterm results of stemless sleeve fixation, they also had an increased risk for malpositioning of the implant [32]. Scior et al. recently reported successful outcomes of a larger series of stemless sleeve constructs [37]. The authors limited its use to cases in which zone 1 and 2 fixation was sufficient (AORI type 1 and type 2A) together with navigation use in cases with femoral or tibial deformity to prevent malpositioning.

The limitations of the present study are its retrospective design, the lack of a comparative group, and the relatively small number of the cohort. The variety of revision indications and using different levels of constraint also constitute limitations. However, the data were prospectively collected, and the procedures were performed by a single surgeon using the same standardised protocol over the study period. Long-term follow-up is still awaited before definitive conclusions are made.

## 5. Conclusion

MS provided excellent outcomes in terms of function, osseointegration, and implant survivorship at the midterm. These successful outcomes were maintained in obese patients with different levels of constraint. Our series supports the use of MS as a viable option in RKA.

## Figures and Tables

**Table 1:** The patients' demographic and clinical parameters.

Parameter	Value
Age (years)	65.4 ± 10.2 (51–98)
Sex	
Male	5 (18.5%)
Female	22 (81.5%)
Side	
R	16 (59.2%)
L	11 (40.8%)
Height (cm)	155 ± 8.8 (142–173)
Weight (kg)	90.2 ± 20.1 (65–119)
BMI (kg/m^2^)	37.6 ± 8.1 (26.7–50.2)
ASA score	
ASA 1	1 (3.7%)
ASA 2	7 (25.9%)
ASA 3	16 (59.3%)
ASA 4	3 (11.1%)
Follow-up (years)	4.1 ± 1.8 (2–7.5)
Time to index revision (years)	9.3 ± 5.6 (0.5–18)

Quantitative variables: mean ± standard deviation; categorical variables: frequency (percentages) with the range in parentheses. TKA = total knee arthroplasty, BMI = body mass index, and ASA = American Society of Anaesthesiologists.

**Table 2:** The details of the RKA and the components implanted.

Parameter	*N* (%)
RKA	27 (100%)
Indications	
Aseptic loosening	14 (51.9%)
PE wear	4 (14.8%)
Infection (second-stage)	3 (11.1%)
Instability	2 (7.4%)
Malalignment	2 (7.4%)
Femoral periprosthetic fracture/nonunion	2 (7.4%)
Metaphyseal sleeves	
All	52 (100%)
Femoral	25 (48.1%)
Tibial	27 (51.9%)
Sleeve size (median)	
Femoral	34 mm (31–46)
Tibial^b^	29 mm (29–61)
75 mm cementless stem^c^	
All	52 (100%)
Diameter (median)	12 mm (10–16)
Component size (median)	
Femoral^d^	2 (1.5–3)
Tibial	1.5 (1.5–3)
PE insert	12.5 mm (10–22.5)
Type of constraint	
TC3-RP	24 (88.9%)
S-ROM Noiles rotating hinge	1 (3.7%)
LPS-DFR	2 (7.4%)

^a^Femoral sleeve sizes are 20 mm, 31 mm, 34 mm, 40 mm, and 46 mm. ^b^Tibial sleeve sizes are 29 mm, 37 mm, 45 mm, 53 mm, and 61 mm. ^c^Two cemented stems implanted with the 2 cases of LPS-DFR. ^d^Femoral component used in 2 cases of LPS-DFR, and 1 case of S-ROM was the smallest size (xx-small). RKA = revision knee arthroplasty, PE = polyethylene, TC3-RB = total condylar III-rotating platform, S-ROM = Sivash-range of motion, and LPS-DFR = limb preservation system-distal femoral replacement.

**Table 3:** The intraoperative bone loss as classified by AORI.

AORI	Femur	Tibia
	*N* (%)	*N* (%)
Type 1	1 (3.7%)	0 (0%)
Type 2A	6 (22.3%)	10 (37%)
Type 2B	17 (62.8%)	13 (48.2%)
Type 3	3 (11.2%)	4 (14.8%)

AORI = Anderson Orthopaedic Research Institute.

## Data Availability

The data used to support the findings of this study are available from the corresponding author upon request.
